# The effectiveness and safety of auricular acupoint-related therapy for nicotine dependence: A systematic review and meta-analysis

**DOI:** 10.18332/tid/200550

**Published:** 2025-02-10

**Authors:** Qindong Mi, Xiaolong Zhao, Zhijun Zhang, Fei Bao

**Affiliations:** 1 Department of Traditional Chinese Medicine, Peking Union Medical College Hospital, Chinese Academy of Medical Sciences and Peking Union Medical College, Beijing, China; 2 Department of Orthopedics, Peking Union Medical College Hospital, Chinese Academy of Medical Sciences and Peking Union Medical College, Beijing, China; 3 Department of Urology Surgery, Peking Union Medical College Hospital, Chinese Academy of Medical Sciences and Peking Union Medical College, Beijing, China

**Keywords:** nicotine dependence, tobacco dependence, acupoint-related therapy, systematic review, meta-analysis, randomized controlled trial

## Abstract

**INTRODUCTION:**

This study aims to systematically evaluate the efficacy and safety of auricular acupuncture-related therapies (AARTs) in managing nicotine dependence (ND).

**METHODS:**

We searched eight databases from their inception through December 2024 and screened randomized controlled trials (RCTs) evaluating AART for ND. The primary outcome was the ND cessation rate. We assessed study quality using the ROB-2 tool and applied the GRADE approach to determine the certainty or quality of of evidence.

**RESULTS:**

Nine RCTs involving 1032 patients were analyzed. Meta-analysis results indicate that AART significantly reduces the Minnesota Nicotine Withdrawal Scale score (MNWS) in ND patients compared to nicotine replacement therapy (NRT) (mean difference, MD=1.47; 95% CI: 0.06–2.88, p<0.05). However, no significant differences were observed in ND point cessation rate, Fagerström test for nicotine dependence score (FTND), Hamilton Anxiety Scale score (HAMA), daily smoking volume, or exhaled CO levels between AART and NRT. Notably, AART was associated with a lower incidence of adverse events compared to NRT (relative risk, RR=0.15; 95% CI: 0.04–0.56, p<0.01). There were also no significant differences in the ND point cessation rate between auricular-plaster therapy (APT), body acupuncture (BA), and the combination of APT and BA.

**CONCLUSIONS:**

AART is effective in improving ND, showing greater efficacy in reducing MNWS and enhanced safety compared to NRT. Given the limited number of studies, the optimal AART regimen remains undetermined. Further research is needed to confirm and refine these findings.

## INTRODUCTION

Smoking is a significant public health issue globally, posing a major threat to human health and social development. Annually, approximately 1 million people in China die from diseases caused by tobacco^1^. The 2018–2019 National Health Literacy Survey reported a smoking rate of 25.1% in China, with 46.5–52.9% of smokers exhibiting nicotine dependence (ND)^2^. ND, a mental and behavioral disorder stemming from prolonged tobacco use^3^, significantly hinders smoking cessation efforts^4^. The 2008 US Clinical Guidelines on Tobacco Use and Dependence recommend first-line smoking cessation treatments^5^, including nicotine-based products such as gums, patches, inhalers, lozenges, nasal sprays, and non-nicotine medications like bupropion and varenicline. However, these treatments can have adverse effects; overactivation of dopamine is linked to increased cardiovascular risk^6^; bupropion may heighten the risk of depression and epilepsy, and impair liver function; varenicline, a selective partial agonist at the α4β2 receptor with effects on the 5-HT3 receptor, may cause digestive and sleep disturbances^7^.

Auriculotherapy, blending traditional Chinese and Western medicine, involves stimulating auricular acupoints externally, usually through acupuncture, to prevent and treat various conditions. Since the 1970s, it has been utilized in treating drug and alcohol dependence and smoking cessation^8^. Contemporary clinical trials indicate that the efficacy of auricular acupoint-related therapy for smoking cessation is comparable to that of NRT, with noted advantages in safety and lower relapse rates. Additionally, it effectively alleviates withdrawal symptoms^9,10^. Despite these benefits, inconsistencies in treatment protocols and diagnostic criteria across studies complicate the assessment of its safety and effectiveness for ND. Therefore, this study employs the Cochrane systematic review method to conduct a meta-analysis of the relevant literature, aiming to provide reliable and effective evidence for treating ND with auricular acupoint-related therapy.

## METHODS

This review was conducted using the Cochrane methodology for meta-analysis^11^. It is reported in accordance with the PRISMA 2020 checklist^12^. The protocol for this review was registered with PROSPERO (CRD42023462049).

### Search strategy

Comprehensive searches were performed in the China Journal Full-Text Database (CNKI), Wan Fang Data Knowledge Service Platform (Wan Fang), VIP Chinese Journal Service Platform (VIP), SinoMed, Embase, Web of Science, Cochrane Library, and PubMed. The search covered the period from database inception to 9 December 2024. The search strategy integrated subject headings and free-text terms. Key search terms included: ‘acupuncture, ear’, ‘auricular acupuncture’, ‘auriculotherapy’, ‘acupressure’, ‘tobacco use’, ‘nicotine addiction’, ‘tobacco dependence’, ‘nicotine dependence’, ‘smoking cessation’, ‘randomized controlled trial’, and ‘randomization’. Detailed search strategies are available in the Supplementary file. Additional methods involved searches by authorship and citation.

### Inclusion criteria

Only RCTs were considered. Individuals included in these trials were required to meet established diagnostic criteria for ND. This encompassed the International Classification of Diseases (ICD-10) criteria for drug dependence^3^, the Diagnostic and Statistical Manual of Mental Disorders, 4th Edition (DSM-IV) criteria for ND^13^, the 2015 Chinese Clinical Smoking Cessation Guidelines for substance dependence^14^, the Chinese Classification of Mental Disorders (CCMD-3)^15^, or a score of ≥4 on the Fagerström test for nicotine dependence scale (FTND)^16^, indicating moderate to severe dependence. Eligibility was irrespective of age, gender, or origin. The main intervention was auricular acupoint-related therapy (AART), including methods such as auricular-plaster therapy (APT), ear acupuncture (EA), auricular buried therapy (ABT), laser auricular acupuncture (LAA), transcutaneous auricular vagus nerve stimulation (Ta-VNS), and combination therapy (CT). Control measures were not restricted. Primary outcomes included ND point cessation rates (both post-treatment and long-term following follow-up), FTND, Minnesota Nicotine Withdrawal Scale score (MNWS), Heaviness of Smoking Index (HSI), Hamilton Anxiety Scale score (HAMA), exhaled carbon monoxide (CO) levels, and daily smoking volume.

### Exclusion criteria

Republished works, animal studies, theoretical research, and dissertations were excluded. Trials were also excluded if participants had severe comorbid diseases other than ND, if the studies were non-randomized, or if the raw data were missing and could not be statistically estimated.

### Data collection process

The literature was screened by two independent researchers using EndNote 20. Each reviewer examined the title, abstract, and full text to identify eligible studies. Following the initial screening, the reviewers exchanged their findings. In case of disagreement, a third-party expert was consulted to resolve any controversies and make a final decision on inclusion. Data extraction included authors, sample size, intervention and control measures, outcome indicators, frequency of treatment, duration of intervention, and adverse reactions. Primary outcome measures extracted were withdrawal rates, daily smoking quantity (cigarettes), exhaled CO levels, and various scale scores.

### Risk of bias assessment

Two independent assessors evaluated the quality and risk of bias in the included studies using the Cochrane Manual for Systematic Reviews on Risk of Bias 2 (ROB-2)^17^. Bias was assessed across five domains: randomization process, deviation from the intended interventions, missing outcome data, outcome measurement, and result reporting. These domains were rated as low risk, some concern, or high risk. The overall risk of bias was determined; any disputes were resolved through discussion with a third-party researcher.

### Statistical analysis

Statistical analysis was performed using RevMan 5.4.0 and R-4.4.2 to produce forest and funnel plots. Continuous variables were analyzed using mean difference (MD) and standard deviation (SD). If SD data were missing from the original studies, estimations were made using the methods recommended in the Cochrane Handbook. Data were analyzed using the weighted MD method. Relative risk (RR) was used to analyze count data, with effect sizes presented as 95% confidence intervals (CI). Heterogeneity testing was conducted during the meta-analysis; I^2^ >50% indicated significant heterogeneity, prompting the use of a random-effects model, while I^2^ ≤50% indicated no significant heterogeneity, justifying a fixed-effects model. Sensitivity analysis was performed using the leave-one-out method. Statistical significance was noted when p<0.05. Funnel plots were used to assess publication bias; symmetry in the funnel plot suggested an absence of publication bias, whereas asymmetry indicated potential bias.

### Quality of evidence

The GRADE Profiler (version 3.6) was used to assess the quality of evidence from the included studies. The GRADE approach offers a systematic framework for evaluating the quality of evidence related to the effectiveness of interventions and for formulating clinical recommendations based on this evaluation^18^. The two reviewers independently assessed the evidence quality from the updated meta-analyses and reached a consensus after discussion.

## RESULTS

### Screening

**Figure 1:**
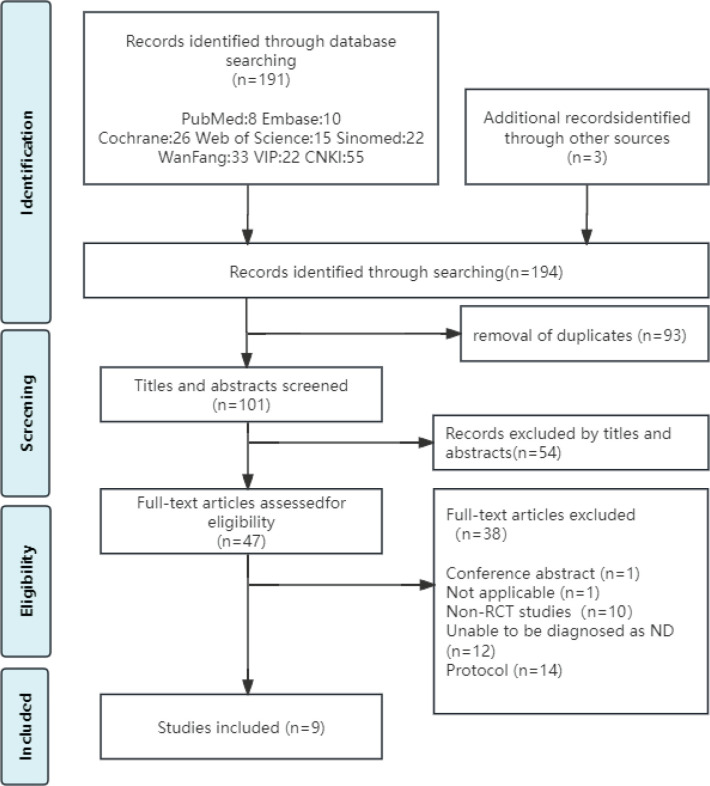
Flowchart of study selection

A total of 194 relevant articles were retrieved, and 9 RCTs were included10,^19-26^. The flowchart of database retrieval is shown in Figure 1.

### Study characteristics

All studies involved patients diagnosed with ND as defined in the Methods section. The total sample size was 1032 cases. The main interventions were ear acupuncture, APT, and ear acupoint combination therapy. The primary control measure was NRT. Key outcomes included ND cessation rate, various scale scores, exhaled CO content, and daily smoking volume. Table 1 details the characteristics of the included RCTs on a population of adult smokers.

**Table 1:** Basic characteristics of the included RCT studies

*Authors Year*	*Sample size*	*Methods of intervention*		*Outcome measures*	*Therapy duration (weeks)*	*Adverse reaction*	*Diagnostic criteria*
		*Trials*	*Controls*				
Aycicegi-Dinn^19^ 2011	47	APT	SA	①②	3	Not reported	FTND ≥5
Chai^20^ 2022	90	T1: APT T2: BA + APT	BA	①②④	8	Not reported	CCSCG (2015)
Cai^21^ 2019	400	T1: APT T2: BA + APT	C1: NRT C2: BA	①②⑥⑦	8	Not reported	ICD-10
Chen^22^ 2023	60	BA + APT	NRT	①②⑤⑥	8	Not reported	CCSCG (2015)
Chen^10^ 2022	200	APT + Ta-VNS	NRT	①②③	8	I: 1 headache C: 5 skin allergies	CCSCG (2015)
Jang^23^ 2019	41	EA + BA + AT + NRT	NRT	①②③	4	Not reported	FTND ≥4
Ji^24^ 2023	64	BA + APT	NRT	①②③⑥⑦	8	I: None C: 5 skin allergies	ICD-10
Liu^25^ 2015	48	BA + APT	APT	①②	4	Not reported	DSM-IV
Zhang^26^ 2022	82	BA + APT	NRT	①⑤⑥⑦	8	I: 1 discomfort C: 4 skin allergies 5 discomfort	CCMD-3

APT: auricular-plaster therapy. SA: sham AART. 3. EA: ear acupuncture. 4. BA: body acupuncture. 5. NRT: nicotine replacement therapy. Ta-VNS: transcutaneous auricular vagus nerve stimulation. AT: aromatherapy. ① Withdrawal rate. ② Fagerström test for nicotine dependence scale score (FTND). ③ Minnesota Nicotine Withdrawal Scale score (MNWS). ④ Smoking Intensity Index scale score (HSI). ⑤ Hamilton Anxiety scale score (HAMA). ⑥ Exhaled carbon monoxide (CO) level. ⑦ Daily smoking volume (cigarettes). CCSCG (2015): Chinese Clinical Smoking Cessation Guidelines (2015 Edition). ICD-10: International Classification of Diseases, 10th Revision. DSM-IV: Diagnostic and Statistical Manual of Mental Disorders, 4th Edition. CCMD-3: Chinese Classification of Mental Disorders (3rd Edition).

### Risk of bias assessment

**Figure 2:**
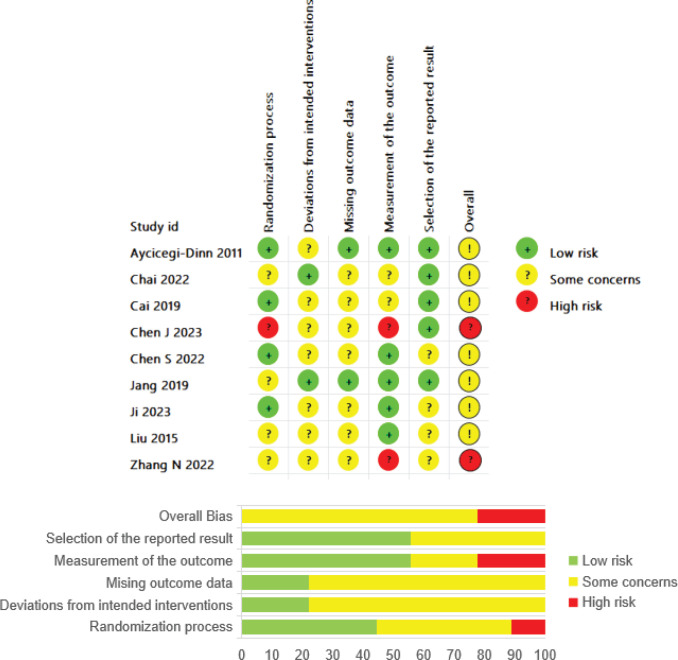
Risk of bias summary of updated meta-analysis about AARTs for ND

The ‘Randomization process’ in some studies was rated as showing ‘some concern’ or ‘high risk’, primarily because these studies either only described the randomization process without mentioning allocation concealment or had issues with allocation concealment. The dimensions of ‘Deviations from intended interventions’ and ‘missing outcome data’ generally showed ‘some concern’. The former was due to the challenges in blinding physicians and patients in some AART studies, and the latter due to issues in data availability and management. In the ‘Measurement of the outcome’ dimension, reliance on self-reported outcomes and subjective scales, or failure to describe the blinding of outcome assessors, raised concerns or indicated a high risk of measurement bias. Most studies exhibited ‘some concerns’ in the ‘Selection of the reported result’ dimension, often due to a lack of study pre-registration. The final evaluations indicated that eight studies had an overall ‘some concerns’ risk rating, and two had an overall ‘high risk’. Figure 2 presents the risk of bias map for the included studies.

### Meta-analysis of the results

*AART versus NRT on ND*

A total of 6 studies^10,20,22-24,26^ analyzed the effects of AART compared to NRT on ND, examining seven different outcomes using the GRADE approach to assess the quality of the updated evidence (Table 2).

**Table 2:** Summary of evidence for ND: AART versus NRT

*Number of studies*	*Design*	*Quality assessment*					*Number of patients*		*Effect*		*Quality of evidence GRADE*	*Importance*
		*Risk of bias*	*Inconsistency*	*Indirectness*	*Imprecision*	*Other considerations*	*AART*	*NRT*	*Relative risk (95% CI)*	*Absolute*		
**ND cessation rate**												
6	RCTs	Not serious	Serious^a^	Serious^b^	Not serious	None	104/421 (24.7%)	115/422 (27.3%)	0.91 (0.73–1.14) p=0.41	25 fewer per 1000 (from 74 fewer to 38 more)	⊕⊕◯◯ Low	Critical
								28%		25 fewer per 1000 (from 76 fewer to 39 more)		
FTND*												
5	RCTs	Not serious	Serious^a^	Serious^b^	Not serious	None	382	383		MD=0.29 higher (0.1 lower to 0.69 higher) p=0.15	⊕⊕◯◯ Low	Important
MNWS*												
3	RCTs	Serious^c^	Not serious	Serious^b^	Not serious	None	152	153		MD=1.47 higher (0.06 to 2.88 higher) p=0.04	⊕⊕◯◯ Low	Important
HAMA*												
2	RCTs	Not serious	Very serious^a,c^	Serious^b^	Not serious	None	71	71		MD=0.32 higher (0.79 lower to 1.43 higher) p=0.57	⊕◯◯◯ Very low	Important
Exhaled CO*												
3	RCTs	Not serious	Very serious^a,c^	Serious^b^	Not serious	None	103	103		MD=0.73 higher (0.53 lower to 1.98 higher) p=0.26	⊕◯◯◯ Very low	Important
Daily smoking volume*												
2	RCTs	Not serious	Very serious^a,c^	Serious^b^	Not serious	None	73	73		MD=0.01 lower (0.94 lower to 0.93 higher) p=0.99	⊕◯◯◯ Very low	Important
**Adverse reactions rate**												
3	RCTs	Not serious	Serious^d^	Serious^b^	Not serious	None	2/241 (0.8%)	16/241 (6.6%)	0.15 (0.04–0.56) p=0.005	56 fewer per 1000 (from 29 fewer to 64 fewer)	⊕⊕◯◯ Low	Critical
								5%		43 fewer per 1000 (from 22 fewer to 48 fewer)		

* Better indicated by lower values.

a The 95% confidence interval for the effect estimate included the null value. No explanation was provided.

b A substantial proportion (2/3) of the evidence base was composed of studies with moderate bias concerns.

c The sample size was insufficient to draw robust conclusions (<400).

d Obvious benefits or harms. MD: mean difference.

*ND point cessation rate*

**Figure 3:**
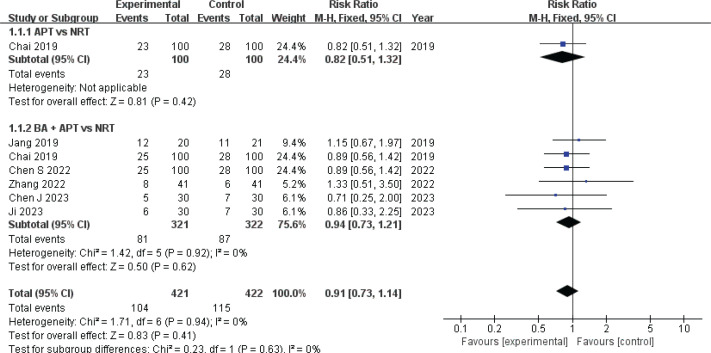
Forest plot of AART versus NRT for ND patients point cessation rate

Six studies^10,20,22-24,26^ evaluated the ND cessation rate using a fixed-effect model (I^2^=0) with 743 patients. The results showed no significant difference in ND cessation rates between the two groups (RR=0.91; 95% CI: 0.73–1.14, p>0.05; low certainty). (Table 2, Figure 3). Subgroup analyses were conducted to compare specific therapies with NRT. One study^20^ found that APT had a lower ND cessation rate compared to NRT (RR=0.82; 95% CI: 0.51–1.32, p<0.05) (Figure 3). Six studies^10,20,22-24^ compared the effects of combination therapy (CT) and NRT on ND cessation rate, finding no statistical significance (RR=0.94; 95% CI: 0.73–1.21, p>0.05) (Figure 3).

*FTND score*

**Figure 4:**
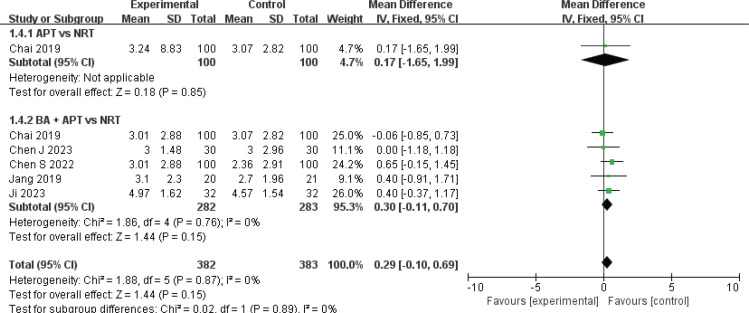
Forest plot of AART versus NRT for ND patients FTND score

Five studies^10,20,22-24^ involving 565 patients used a fixed-effect model to analyze the impact of AART and NRT on FTND scores (I^2^=0%). The results indicated no significant difference in FTND scores between the groups (MD=0.29; 95% CI: -0.10–0.69, p>0.05; low certainty) (Table 2, Figure 4). Subgroup analysis revealed no significant differences in FTND scores between APT and NRT (MD=0.17; 95% CI: -1.65–1.99, p>0.05) and between CT and NRT (MD=0.3; 95% CI: -0.11–0.70, p>0.05) (Figure 4).

*MNWS score*

Three studies^10,23,24^ analyzed the effects of AART and NRT on MNWS scores, involving 305 patients. A fixed-effect model analysis was used (I^2^=0%), and the results indicated that AART was more effective in reducing MNWS scores compared to NRT (MD=1.47; 95% CI: 0.06–2.88, p<0.05; low certainty) (Table 2, Supplementary file Figure 1).

*HAMA score*

Two studies^22,26^ involving 142 patients analyzed the effects of AART and NRT on HAMA scores using a fixed-effect model (I^2^=0%). The results showed no statistically significant difference (MD=0.32; 95% CI: -0.79–1.43, p>0.05; very low certainty) (Table 2, Supplementary file Figure 2).

*Exhaled CO*

Three studies^22,24,26^ involving 206 patients analyzed the effects of AART and NRT on exhaled CO using a fixed-effect model (I^2^=0%). The results showed no statistically significant difference (MD=0.73; 95% CI: -0.53–1.98, p>0.05; very low certainty) (Table 2, Supplementary file Figure 3).

*Daily smoking volume*

Two studies^24,26^ analyzed the effects of AART and NRT on daily smoking volume in 146 patients. A fixed-effect model was used (I^2^=0%), and the results showed no statistically significant difference (MD= -0.01; 95% CI: -0.94–0.93, p>0.05; low certainty; very low certainty] (Table 2, Supplementary file Figure 4).

*Adverse reactions rate*

Three studies^10,24,26^ involving 482 patients recorded the adverse reactions of AART compared to NRT. A fixed-effect model was used (I^2^=0%), and the results showed that the incidence of adverse reactions was lower with AART than with NRT (RR=0.15; (95% CI: 0.04–0.56, p<0.01; low certainty) (Table 2, Supplementary file Figure 5).

### APT, BA, APT combined with BA, pairwise comparison

Data from three studies^20,21,25^ were extracted to compare the ND cessation rates of APT, BA, and APT combined with BA, both as combined therapies or alone. Meta-analysis showed no statistical significance in these comparisons (Table 3, Supplementary file Figure 6).

**Table 3:** APT, BA, APT combined with BA, pairwise comparison

*Methods of intervention*		*Number of studies*	*Sample size*	*RR*	*95% CI*	*p*
*Trials*	*Controls*					
APT	BA	2 [20,21]	260	1.06	(0.70–1.63)	0.77
APT + BA	APT	3 [20,21,25]	460	1.24	(0.88–1.74)	0.21
APT + BA	BA	2 [20,21]	260	1.19	(0.73–2.23)	0.40

RR: relative risk.

### Sensitivity analyses and publication bias

Leave-one-out sensitivity analyses were performed for outcomes including more than five studies, and the results were robust (Supplementary file Figures 6–7). The funnel plots were largely symmetrical, indicating no significant publication bias (Supplementary file Figures 8–9).

## DISCUSSION

### Findings from systematic reviews

The results of this study indicate that AART is potentially more effective in reducing MNWS scores compared to NRT. However, for other outcomes, there is no statistically significant difference between AART and NRT. Despite the limited number and quality of studies, there is insufficient evidence to conclusively determine that AART is superior to NRT. Nevertheless, it is evident that AART is associated with fewer adverse effects. NRT often causes irritation at the site of application and may lead to non-ischemic chest pain and palpitations^27^, which can contribute to the failure of smoking cessation treatments. Thus, the safety profile of AART suggests its potential as a new first-line therapy for ND.

The current study found no statistically significant difference in the effectiveness of APT and BA, either alone or in combination, for treating ND. However, previous studies^23,28,29^ have indicated that the combination of APT and BA is more effective than either therapy alone for smoking cessation, necessitating further validation in future research. Presently, research on auricular point treatment for ND primarily involves clinical studies focusing on point selection and effectiveness^30^, with its underlying mechanisms still in preliminary stage. The mechanism of BA in smoking cessation may involve inducing the body to produce a large number of endogenous opioids, which bind to central opioid receptors, thereby alleviating the comprehensive withdrawal symptoms caused by nicotine cessation^31^. Whether auricular acupoints exert a similar therapeutic effect on ND through this mechanism merits further investigation.

### AART versus CBT and sham AART controversy

In addition to pharmacological treatments, cognitive behavioral therapy (CBT) is an effective intervention for ND^32^. Although high-quality RCTs comparing AART with CBT are lacking, our analysis of four RCTs showed that combining auriculotherapy with CBT^29,33,34^ was more effective than CBT alone in improving smoking cessation rates (RR=1.72; 95% CI: 1.20–2.47, p<0.01) (Supplementary file Figures 8–9). This finding underscores AART’s potential in ND treatment.

The use of sham acupuncture, whether auricular or body, in control groups remains controversial^35^. Only one of the studies included used sham AART (SA) as a control, preventing further analysis in this review^19^. We identified five RCTs that employed SA; two of these used ineffective auricular stimulation^19,29,36-38^, and three used acupuncture points deemed therapeutically inactive by researchers^19,36^. The efficacy of ‘ineffective stimulation’ versus ‘inactive points’ as true controls continues to be debated^29,37,38^, leading to varying placebo effects and conflicting study results. Future studies should carefully select sham acupuncture or placebo methods to reduce potential biases and inaccuracies in research findings.

### Comparisons with other studies

Past systematic reviews of acupuncture for treating tobacco withdrawal syndromes and ND^31,39,40^ did not rigorously exclude studies lacking proper ND diagnostic criteria, potentially causing significant heterogeneity among the studies. Furthermore, these reviews did not specifically focus on AART, nor did they provide a detailed evaluation of AART. This led to an incomplete assessment of AART’s efficacy and safety in treating ND. Our study included only RCTs that adhered to clear diagnostic criteria for ND and concentrated on AART, conducting detailed subgroup analyses and evaluating multiple outcomes using the GRADE approach.

### Strengths and limitations

This review systematically evaluates the efficacy and safety of AART in improving ND. We focused on AART and assessed the evidence quality for its effects on various outcomes using the GRADE approach. The limitations, according to PRISMA guidelines, include a limited number of studies, which restricts our ability to determine the optimal treatment regimen. Variability in diagnostic criteria across studies complicates the evaluation of AART’s safety and effectiveness. The small sample sizes may affect the stability and reliability of the findings. Moreover, the analysis does not extensively cover the long-term effects of AART on ND. Potential biases arise from inadequate handling of missing data and the possibility of selective reporting. Many studies lacked blinding of participants or interveners, introducing performance bias. These issues underscore the need for more high-quality research in this field.

### Implications

This study reviews current evidence from RCTs on AART for ND treatment. The findings suggest that AART has a lower incidence of adverse effects compared to NRT. Auriculotherapy is advantageous due to its safety, cost-effectiveness, ease of use, and compatibility with other treatments. The study preliminarily demonstrates AART’s significant potential in treating ND and emphasizes the need for additional high-quality experiments to further define the optimal AART regimen and enhance the findings of this research.

## Supplementary Material



## Data Availability

Data sharing is not applicable to this article as no new data were created.
