# Supplementary material for: The effectiveness and safety of auricular acupoint-related therapy for nicotine dependence: A systematic review and meta-analysis
Source: Tob Induc Dis. 2025 Feb 10;23:10.18332/tid/200550. doi: 10.18332/tid/200550 (PMC11808481; doi:10.18332/tid/200550)
Supplement: Supplementary file 1 [file TID-23-15-s1.pdf]

Supplementary file： The effectiveness and safety of auricular acupoint-related therapy on Nicotine Dependence: A systematic review and meta-analysis

1.Search strategy 2
(1) PUBMED:8 2
(2) WOS:15 2
(3) EMBASE:10 3
(4) Cochrane： 26 3
(5) CNKI： 55 5
(7) Wangfang： 33 6
(8) Sinomed： 22 6
2. Meta-analysis of forest plots 7
(1) Figure 1. Forest plot of AART vs NRT for ND patients MNWS score. 7
(2) Figure 2. Forest plot of AART vs NRT for ND patients HAMA score. 7
(3) Figure 3. Forest plot of AART vs NRT for ND patients Exhaled CO. 8
(4) Figure 4. Forest plot of AART vs NRT for ND patients daily smoking volume. 8
(5) Figure 5. Forest plot of AART vs NRT for ND patients adverse reactions rate. 8
3. Meta-analysis of leave-one-out plots 9
(1) Figure 6. Leave-one-out plots of AART vs NRT for ND patients point cessation rate. 9
(2) Figure 7. Leave-one-out plots of AART vs NRT for ND patients FTND score. 9
4. Meta-analysis of funnel plots 10
(1) Figure 8. Funnel plots of AART vs NRT for ND patients point cessation rate. 10
(2) Figure 9. Funnel plots of AART vs NRT for ND patients FTND score. 10
5. AART versus CBT and sham AART controversy 11
(1) Figure 10. Forest plots of AART vs NRT for smokers point cessation rate. 11
(1) Figure 11. Funnel plots of AART vs NRT for smokers point cessation rate. 11

1.Search strategy

（1） PUBMED:8

((((((((((((((((\"Tobacco Use Disorder\"[Mesh]) OR (Disorder, Tobacco Use[Title/Abstract])) OR (Tobacco Use Disorders[Title/Abstract])) OR (Tobacco-Use Disorder[Title/Abstract])) OR (Disorder, Tobacco-Use[Title/Abstract])) OR (Nicotine Addiction[Title/Abstract])) OR (Addiction, Nicotine[Title/Abstract])) OR (Nicotine Addictions[Title/Abstract])) OR (Tobacco Dependence[Title/Abstract])) OR (Dependence, Tobacco[Title/Abstract])) OR (Nicotine Use Disorder[Title/Abstract])) OR (Disorder, Nicotine Use[Title/Abstract])) OR (Nicotine Use Disorders[Title/Abstract])) OR (Nicotine Dependence[Title/Abstract])) OR (Dependence, Nicotine[Title/Abstract]))) AND (((((((((((((((((((((\"Acupuncture, Ear\"[Mesh]) OR (Acupunctures, Ear[Title/Abstract])) OR (Ear Acupunctures[Title/Abstract])) OR (Auricular Acupuncture[Title/Abstract])) OR (Ear Acupuncture[Title/Abstract])) OR (Acupuncture, Auricular[Title/Abstract])) OR (Acupunctures, Auricular[Title/Abstract])) OR (Auricular Acupunctures[Title/Abstract])) OR (Battlefield Acupuncture[Title/Abstract])) OR (Auriculotherapy[Title/Abstract])) OR (Auricular Therapy[Title/Abstract])) OR (Ear Massage[Title/Abstract])) OR (Auricular Point Sticking[Title/Abstract])) OR (Auricular Point Pressing[Title/Abstract])) OR (Ears Pressure Beans[Title/Abstract])) OR (Transcutaneous Auricular Vagus Nerve Stimulation[Title/Abstract])) OR (ta-VNS[Title/Abstract])) OR (Ear Point Moxibustion[Title/Abstract])) OR (Ear Point Bloodletting[Title/Abstract])) OR (Ear Point Injection[Title/Abstract])) OR (Acupressure[Title/Abstract])) AND (randomizedcontrolledtrial[Filter]) AND ((randomizedcontrolledtrial[Filter]) AND (1960/6/20:2024/12/9[pdat]))

（2） WOS:15

((TS=(Tobacco Use Disorder) OR AB=(Tobacco Use Disorder OR Disorder, Tobacco Use OR Tobacco Use Disorders OR Tobacco-Use Disorder OR Disorder, Tobacco-Use OR Nicotine Addiction OR Addiction, Nicotine OR Nicotine Addictions OR Tobacco Dependence OR Dependence, Tobacco OR Nicotine Use Disorder OR Disorder, Nicotine Use OR Nicotine Use Disorders OR Nicotine Dependence OR Dependence, Nicotine)) AND (TS=(Acupuncture, Ear) OR AB=(Acupunctures, Ear OR Ear Acupunctures OR Auricular Acupuncture OR Ear Acupuncture OR Acupuncture, Auricular OR Acupunctures, Auricular OR Auricular Acupunctures OR Battlefield Acupuncture OR Auriculotherapy OR Auricular Therapy OR Ear Massage OR Auricular Point Sticking OR Auricular Point Pressing OR Ears Pressure Beans OR Transcutaneous Auricular Vagus Nerve Stimulation OR ta-VNS OR Ear Point Moxibustion OR Ear Point Bloodletting OR

Ear Point Injection OR Acupressure OR Auricular acupressure)) AND (TS=(Randomized Controlled Trial) OR AB=(Randomized Controlled Trial OR Randomized OR Random Allocation OR Clinical Trials, Randomized OR Trials, Randomized Clinical OR Controlled Clinical Trials, Randomized OR RCT))) AND DOP=(1960-06-20/2024-12-09)

（3） EMBASE:10

('tobacco use disorder':ti,ab,kw OR 'disorder, tobacco use':ti,ab,kw OR 'tobacco use disorders':ti,ab,kw OR 'tobacco-use disorder':ti,ab,kw OR 'disorder, tobacco-use':ti,ab,kw OR 'nicotine addiction':ti,ab,kw OR 'addiction, nicotine':ti,ab,kw OR 'nicotine addictions':ti,ab,kw OR 'tobacco dependence':ti,ab,kw OR 'dependence, tobacco':ti,ab,kw OR 'nicotine use disorder':ti,ab,kw OR 'disorder, nicotine use':ti,ab,kw OR 'nicotine use disorders':ti,ab,kw OR 'nicotine dependence':ti,ab,kw OR 'dependence, nicotine':ti,ab,kw) AND ('acupunctures, ear':ti,ab,kw OR 'ear acupunctures':ti,ab,kw OR 'auricular acupuncture':ti,ab,kw OR 'ear acupuncture':ti,ab,kw OR 'acupuncture, auricular':ti,ab,kw OR 'acupunctures, auricular':ti,ab,kw OR 'auricular acupunctures':ti,ab,kw OR 'battlefield acupuncture':ti,ab,kw OR auriculotherapy:ti,ab,kw OR 'auricular therapy':ti,ab,kw OR 'ear massage':ti,ab,kw OR 'auricular point sticking':ti,ab,kw OR 'auricular point pressing':ti,ab,kw OR 'ears pressure beans':ti,ab,kw OR 'transcutaneous auricular vagus nerve stimulation':ti,ab,kw OR 'ear point moxibustion':ti,ab,kw OR 'ear point bloodletting':ti,ab,kw OR 'ear point injection':ti,ab,kw OR 'ta vns':ti,ab,kw OR ' acupressure':ti,ab,kw OR ' auricular acupressure':ti,ab,kw) AND [randomized controlled trial]/lim AND [<1966-2024]/py AND [20-06-1960]/sd NOT [09-12-2024]/sd

（4） Cochrane： 26

#1 MeSH descriptor: [Tobacco Use Disorder] explode all trees 2246

#2 (Tobacco Use Disorder OR Disorder, Tobacco Use OR Tobacco Use Disorders OR Tobacco-Use Disorder OR Disorder, Tobacco-Use OR Nicotine Addiction OR Addiction, Nicotine OR Nicotine Addictions OR Tobacco Dependence OR Dependence, Tobacco OR Nicotine Use Disorder OR Disorder, Nicotine Use OR Nicotine Use Disorders OR Nicotine Dependence OR Dependence, Nicotine):ti,ab,kw (Word variations have been searched) 7371

#3 #1 OR #2 7371

#4 MeSH descriptor: [Acupuncture, Ear] explode all trees 271

#5 (Acupunctures, Ear OR Ear Acupunctures OR Auricular Acupuncture OR Ear Acupuncture OR Acupuncture, Auricular OR Acupunctures, Auricular OR Auricular Acupunctures OR Battlefield Acupuncture OR Auriculotherapy OR Auricular Therapy OR

Ear Massage OR Auricular Point Sticking OR Auricular Point Pressing OR Ears Pressure Beans OR Transcutaneous Auricular Vagus Nerve Stimulation OR ta-VNS OR Ear Point Moxibustion OR Ear Point Bloodletting OR Ear Point Injection OR Acupressure OR Auricular acupressure):ti,ab,kw (Word variations have been searched) 4931

#6 #4 OR #5 4931

#7 MeSH descriptor: [Randomized Controlled Trials as Topic] explode all trees58315

#8 (Randomized Controlled Trial OR Randomized OR Random Allocation OR Clinical Trials, Randomized OR Trials, Randomized Clinical OR Controlled Clinical Trials, Randomized OR RCT):ti,ab,kw (Word variations have been searched) 1397444

#9 #7 OR #8 1397563

#10 #3 AND #6 AND #9 with Cochrane Library publication date Between Jan 1000 and Dec 2024 26

（5）CNKI：55

（主题：尼古丁依赖 + 烟草依赖 + 戒烟 + 戒除烟瘾 + 烟瘾 + 烟草成瘾 + 烟草截断 + 尼古丁戒断）OR（篇关摘：尼古丁依赖 + 烟草依赖 + 戒烟 + 戒除烟瘾 + 烟瘾 + 烟草成瘾 + 烟草截断 + 尼古丁戒断(精确)）OR（关键词：尼古丁依赖 + 烟草依赖 + 戒烟 + 戒除烟瘾 + 烟瘾 + 烟草成瘾 + 烟草截断 + 尼古丁戒断(精确)）AND（主题：耳穴 + 耳穴贴压 + 耳针疗法 + 耳穴疗法 + 耳豆贴压 +耳穴压豆 + 耳豆 + 耳针疗法 + 耳甲迷走神经 + 耳皮迷走神经 +经皮耳穴迷走神经 + 耳灸 + 耳穴放血 + 耳穴按摩 + 耳穴注射 + 熨耳法 + 耳穴刮痧 + 耳穴）OR（篇关摘：耳穴 + 耳穴贴压 + 耳针疗法 + 耳穴疗法 + 耳豆贴压 +耳穴压豆 + 耳豆 + 耳针疗法 + 耳甲迷走神经 + 耳皮迷走神经 +经皮耳穴迷走神经 + 耳灸 + 耳穴放血 + 耳穴按摩 + 耳穴注射 + 熨耳法 + 耳穴刮痧 + 耳穴(精确)）OR（关键词：耳穴 + 耳穴贴压 + 耳针疗法 + 耳穴疗法 + 耳豆贴压 +耳穴压豆 + 耳豆 + 耳针疗法 + 耳甲迷走神经 + 耳皮迷走神经 +经皮耳穴迷走神经 + 耳灸 + 耳穴放血 + 耳穴按摩 + 耳穴注射 + 熨耳法 + 耳穴刮痧 + 耳穴(精确)）AND（主题：随机对照研究 + RCT + 随机对照试验 + 随机对照 + 随机 + 随机分配）OR（篇关摘：随机对照研究 + RCT + 随机对照试验 + 随机对照 + 随机 + 随机分配(精确)）OR（关键词：随机对照研究 + RCT + 随机对照试验 + 随机对照 + 随机 + 随机分配(精确)）AND（主题：随机对照研究 + RCT + 随机对照试验 + 随机对照 + 随机 + 随机分配）OR（篇关摘：随机对照研究 + RCT + 随机对照试验 + 随机对照 + 随机 + 随机分配(精确)）OR（关键词：随机对照研究 + RCT + 随机对照试验 + 随机对照 + 随机 + 随机分配(精确)）

（6）VIP：22

检索式：((((((((M=尼古丁依赖 OR M=烟草依赖) OR M=戒烟) OR M=戒除烟瘾) OR M=烟瘾) OR M=烟草成瘾) OR M=烟草截断) OR M=尼古丁戒断) OR (((((((R=尼古丁依赖 OR R=烟草依赖) OR R=戒烟) OR R=戒除烟瘾) OR R=烟瘾) OR R=烟草成瘾) OR R=烟草截断) OR R=尼古丁戒断)) AND ((((((((((((((((M=耳穴 OR M=耳穴贴压) OR M=耳针疗法) OR M=耳穴疗法) OR M=耳豆贴压) OR M=耳穴压豆) OR M=耳豆) OR M=耳针疗法) OR M=耳甲迷走神经) OR M=耳皮迷走神经) OR M=经皮耳穴迷走神经) OR M=耳灸) OR M=耳穴放血) OR M=耳穴按摩) OR M=耳穴注射) OR M=熨耳法) OR M=耳穴刮痧) OR (((((((((((((((R=耳穴 OR R=耳穴贴压) OR R=耳针疗法) OR R=耳穴疗法) OR R=耳豆贴压) OR R=耳穴压豆) OR R=耳豆) OR R=耳针疗法) OR R=耳甲迷走神经) OR R=耳

皮迷走神经) OR R=经皮耳穴迷走神经) OR R=耳灸) OR R=耳穴放血) OR R=耳穴按摩) OR R=耳穴注射) OR R=熨耳法) OR R=耳穴刮痧)) AND ((((((M=随机对照研究 OR M=RCT) OR M=随机对照试验) OR M=随机对照) OR M=随机) OR M=随机分配) OR (((((R=随机对照研究 OR R=RCT) OR R=随机对照试验) OR R=随机对照) OR R=随机) OR R=随机分配)))

（7）Wangfang：33

(((主题:(尼古丁依赖 OR 烟草依赖 OR 戒烟 OR 戒除烟瘾 OR 烟瘾 OR 烟草成瘾 OR 烟草截断 OR 尼古丁戒断) or 题名或关键词:(尼古丁依赖 OR 烟草依赖 OR 戒烟 OR 戒除烟瘾 OR 烟瘾 OR 烟草成瘾 OR 烟草截断 OR 尼古丁戒断) or 关键词:(尼古丁依赖 OR 烟草依赖 OR 戒烟 OR 戒除烟瘾 OR 烟瘾 OR 烟草成瘾 OR 烟草截断 OR 尼古丁戒断)) and (主题:(耳穴 OR 耳穴贴压 OR 耳针疗法 OR 耳穴疗法 OR 耳豆贴压 OR 耳穴压豆 OR 耳豆 OR 耳针疗法 OR 耳甲迷走神经 OR 耳皮迷走神经 OR 经皮耳穴迷走神经 OR 耳灸 OR 耳穴放血) or 题名或关键词:(耳穴 OR 耳穴贴压 OR 耳针疗法 OR 耳穴疗法 OR 耳豆贴压 OR 耳穴压豆 OR 耳豆 OR 耳针疗法 OR 耳甲迷走神经 OR 耳皮迷走神经 OR 经皮耳穴迷走神经 OR 耳灸 OR 耳穴放血) or 关键词:(耳穴 OR 耳穴贴压 OR 耳针疗法 OR 耳穴疗法 OR 耳豆贴压 OR 耳穴压豆 OR 耳豆 OR 耳针疗法 OR 耳甲迷走神经 OR 耳皮迷走神经 OR 经皮耳穴迷走神经 OR 耳灸 OR 耳穴放血)) and (主题:(随机对照研究 OR RCT OR 随机对照试验 OR 随机对照 OR 随机 OR 随机分配) or 题名或关键词:(随机对照研究 OR RCT OR 随机对照试验 OR 随机对照 OR 随机 OR 随机分配) or 关键词:(随机对照研究 OR RCT OR 随机对照试验 OR 随机对照 OR 随机 OR 随机分配)))) and 发表时间:1900-2024

（8）Sinomed：22

(("耳穴"[标题:智能] OR "耳穴贴压"[标题:智能] OR "耳针疗法"[标题:智能] OR "耳穴疗法"[标题:智能] OR "耳豆贴压 OR 耳穴压豆"[标题:智能] OR "耳豆"[标题:智能] OR "耳针疗法"[标题:智能] OR "耳甲迷走神经"[标题:智能] OR "耳皮迷走神经 OR 经皮耳穴迷走神经"[标题:智能] OR "耳灸"[标题:智能] OR "耳穴放血"[标题:智能] OR "耳穴按摩"[标题:智能] OR "耳穴注射"[标题:智能] OR "熨耳法"[标题:智能] OR "耳穴刮痧"[标题:智能] OR "耳穴按压"[标题:智能]) OR("耳穴"[摘要:智能] OR "耳穴贴压"[摘要:智能] OR "耳针疗法"[摘要:智能] OR "耳穴疗法"[摘要:智能] OR "耳豆贴压 OR 耳穴压豆"[摘要:智能] OR "耳豆"[摘要:智能] OR "耳针疗法"[摘要:智能] OR "耳甲迷走神经"[摘要:智能] OR "耳皮迷走神经 OR 经皮耳穴迷走神经"[摘要:智能] OR "耳灸"[摘要:智能] OR "耳穴放血"[摘要:智能] OR "耳穴按摩"[摘要:智能] OR "耳穴注射"[摘要:智能] OR "熨耳法"[摘要:智能] OR "耳穴刮痧"[摘要:智能] OR "耳穴按压"[摘要:智能]) OR("耳穴"[常用字段:智能] OR "耳穴贴压"[常用字段:智能] OR "耳针疗法"[常用字段:智能] OR "耳穴疗法"[常用字段:智能] OR "耳豆贴压 OR 耳穴压豆"[常用字段:智能] OR "耳豆"[常用字段:智能] OR "耳针疗法"[常用字段:智能] OR "耳甲迷走神经"[常用字段:智能] OR "耳皮迷走神经 OR 经皮耳穴迷走神经"[常用字段:智能] OR "耳灸"[常用字段:智能] OR "耳穴放血"[常用字段:智能] OR "耳穴按摩"[常用字段:智能] OR "耳穴注射"[常用字段:智能] OR "熨耳法"[常用字段:智能] OR "耳穴刮痧"[常用字段:智能] OR "耳穴按压"[常用字段:智能])) AND (("随机对照研究"[标题:

智能] OR "RCT"[标题:智能] OR "随机对照试验"[标题:智能] OR "随机对照"[标题:智能] OR "随机"[标题:智能] OR "随机分配"[标题:智能]) OR("随机对照研究"[摘要:智能] OR "RCT"[摘要:智能] OR "随机对照试验"[摘要:智能] OR "随机对照"[摘要:智能] OR "随机"[摘要:智能] OR "随机分配"[摘要:智能]) OR("随机对照研究"[全部字段:智能] OR "RCT"[全部字段:智能] OR "随机对照试验"[全部字段:智能] OR "随机对照"[全部字段:智能] OR "随机"[全部字段:智能] OR "随机分配"[全部字段:智能])) AND (("尼古丁依赖"[标题:智能] OR "烟草依赖"[标题:智能] OR "戒烟"[标题:智能] OR "戒除烟瘾"[标题:智能] OR "烟瘾"[标题:智能] OR "烟草成瘾"[标题:智能] OR "烟草截断"[标题:智能] OR "尼古丁戒断"[标题:智能]) OR("尼古丁依赖"[摘要:智能] OR "烟草依赖"[摘要:智能] OR "戒烟"[摘要:智能] OR "戒除烟瘾"[摘要:智能] OR "烟瘾"[摘要:智能] OR "烟草成瘾"[摘要:智能] OR "烟草截断"[摘要:智能] OR "尼古丁戒断"[摘要:智能]) OR("尼古丁依赖"[常用字段:智能] OR "烟草依赖"[常用字段:智能] OR "戒烟"[常用字段:智能] OR "戒除烟瘾"[常用字段:智能] OR "烟瘾"[常用字段:智能] OR "烟草成瘾"[常用字段:智能] OR "烟草截断"[常用字段:智能] OR "尼古丁戒断"[常用字段:智能]))

限定条件： 1900-2024;

2. Meta-analysis of forest plots

(1) Figure 1. Forest plot of AART vs NRT for ND patients MNWS score.

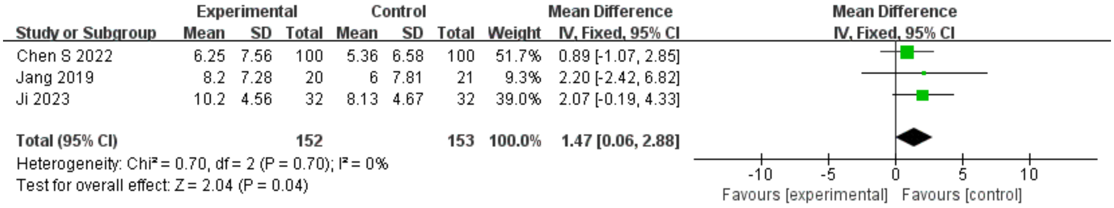

(2) Figure 2. Forest plot of AART vs NRT for ND patients HAMA score.

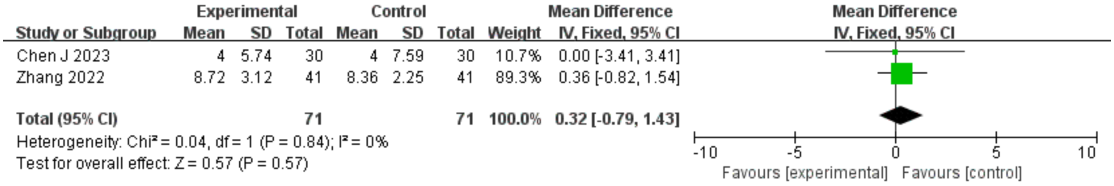

(3) Figure 3. Forest plot of AART vs NRT for ND patients Exhaled CO.

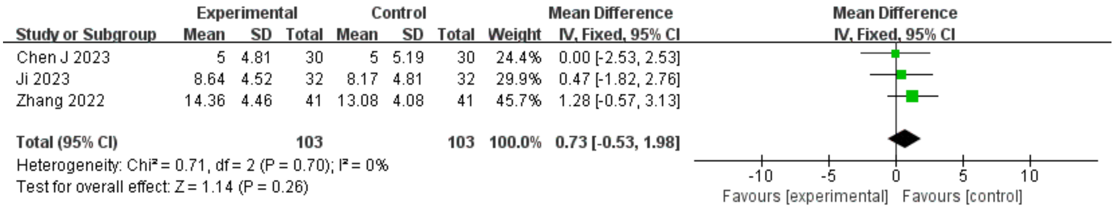

(4) Figure 4. Forest plot of AART vs NRT for ND patients daily smoking volume.

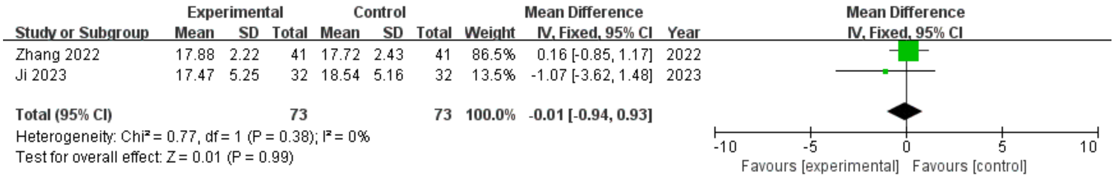

(5) Figure 5. Forest plot of AART vs NRT for ND patients adverse reactions rate.

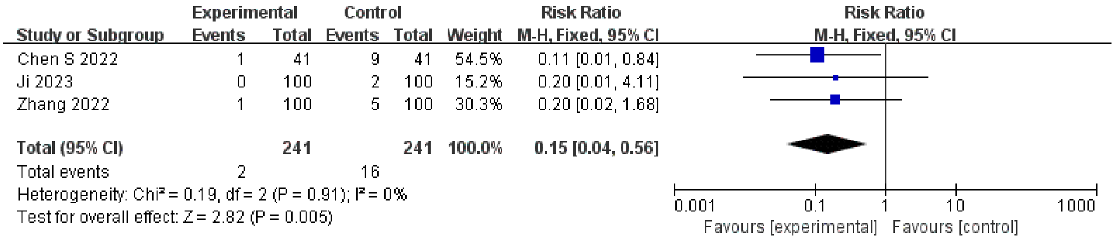

3. Meta-analysis of leave-one-out plots

(1) Figure 6. Leave-one-out plots of AART vs NRT for ND patients point cessation rate.

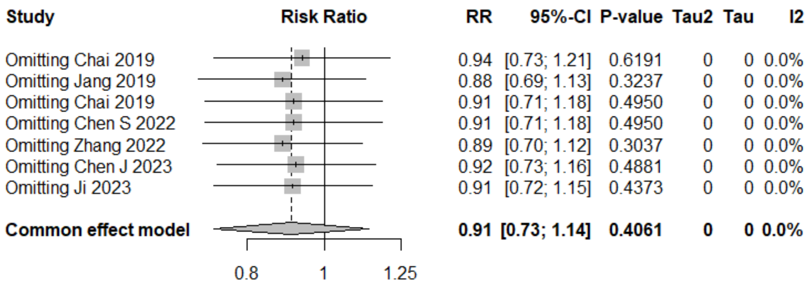

(2) Figure 7. Leave-one-out plots of AART vs NRT for ND patients FTND score.

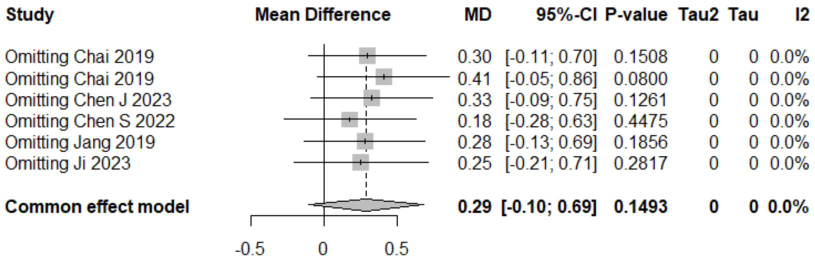

4. Meta-analysis of funnel plots

(1) Figure 8. Funnel plots of AART vs NRT for ND patients point cessation rate.

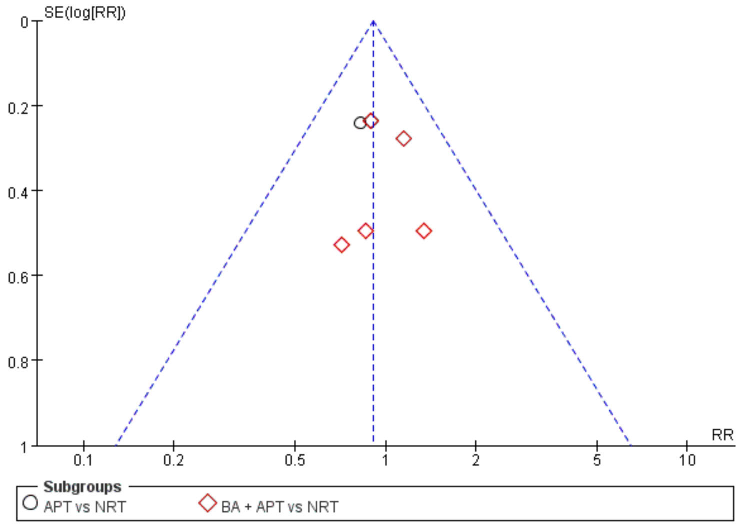

(2) Figure 9. Funnel plots of AART vs NRT for ND patients FTND score.

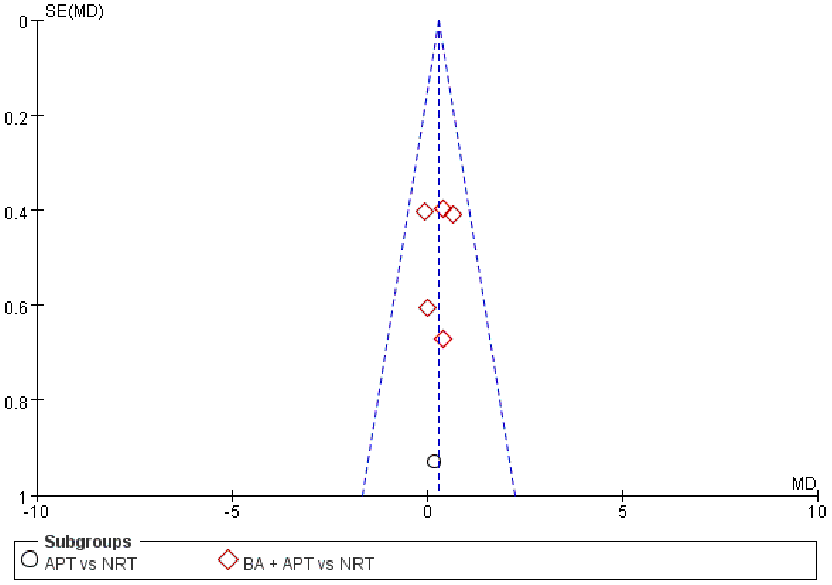

5. AART versus CBT and sham AART controversy

(1) Figure 10. Forest plots of AART vs NRT for smokers point cessation rate.

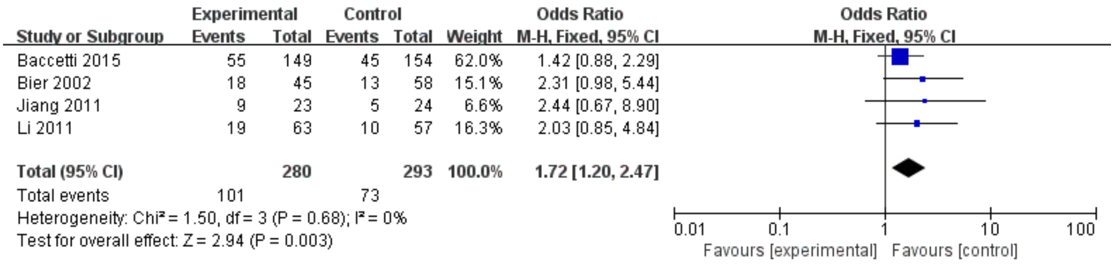

(1) Figure 11. Funnel plots of AART vs NRT for smokers point cessation rate.

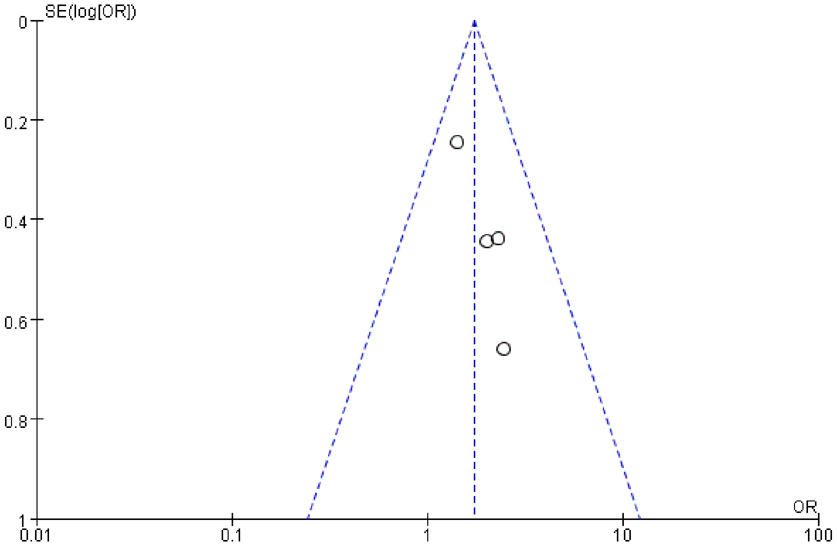

© 2025 Mi Q. et al.
